# Exploring the reclamation pathway science of Beachwood powder for pharmaceutical acetaminophen drug effluent management

**DOI:** 10.1371/journal.pone.0309552

**Published:** 2024-10-11

**Authors:** Ehssan Ahmed Hassan, Maha Tony, Mohamed M. Awad

**Affiliations:** 1 Department of Biology, College of Science and Humanities, Prince Sattam bin Abdul Aziz University, Alkharj, Saudi Arabia; 2 Department of Zoology, Faculty of Science, Suez Canal University, Ismailia, Egypt; 3 Basic Engineering Science Department, Faculty of Engineering, Menoufia University, Shebin El-Kom, Egypt; 4 Advanced Materials/Solar Energy and Environmental Sustainability (AMSEES) Laboratory, Faculty of Engineering, Menoufia University, Shebin El-Kom, Egypt; 5 Department of Mathematics, College of Science and Humanities in Al-Kharj, Prince Sattam bin Abdulaziz University, Al-Kharj, Saudi Arabia; 6 Department of Mathematics, Faculty of Science, Suez Canal University, El-Sheik Zayed, Ismailia, Egypt; Jazan University, SAUDI ARABIA

## Abstract

High effective low-cost substance derived from agriculture-based waste towards a circular economy concept showed a significant green approach for pharmaceuticals uptake in aqueous solution. *Beachwood* sawdust was used as the source of cellulose based adsorbents. Cellulose is isolated from the waste and in parallel magnetite nanoparticles are prepared by the simple co-precipitation technique and the two substances are mixed in various proportions to be acetaminophen adsorbent. Characteristics of the prepared magnetite (M)/sawdust (SD) composite in various proportions (M:SD (1:1), M:SD (1:2), M:SD (1:3), M:SD (1:5) and M:SD (2:1) were assessed using scanning electron microscope (SEM) transmission electron microscope (TEM) and X-ray diffractometer (XRD) which revealed the presence magnetite and cellulose. Also, for the object of recoverable adsorbent, vibrating sample magnetometer (VSM) of the adsorbent is investigated to evaluate its sustainability. The highest removal rate was associated with M:SD (1:2) compared to the other composites and the pristine magnetite or sawdust materials within 2 hours of isotherm time. The adsorption parameters are optimized and the maximal yield is attained at pH (7.0), adsorbent dose of 2.0 g/L at room temperature. The adsorption matrix is following Langmuir model and fitted to the second-order kinetic model. The process is exothermic in nature and highlighted physisorption tendency. The highest monolayer adsorption uptake was investigated at 7.0 mg/g which corresponds to the M:SD (1:2) adsorbent.

## 1. Introduction

Currently, the global trend of the researchers is to convert for a sustainable future through the circular economy approach. Hence, water contamination management is essential technology through the worldwide [1, 2]. Currently, thousands of pharmaceutical products that are signified as emerging pollutants are produced and available in the market [3–5]. However, through their production a massive amount of wastewater is generated and such release onto the environment causing deterioration in the ecosystem [6]. Acetaminophen drug is signified as one of the widely spread in the market and their extensive handling is due to its suggestive therapeutic effectiveness with economic cost. Therefore, the expired stock, the hospital and domestic waste discharge or the industrial wastewater spills is causing their leak into the environment [4]. Such drug is detected in numerous ecological environments since its imperfect metabolism and mineralization [7–9]. The release of acetaminophen onto surface and underground waters causes a sever human health destruction on hormone disruption, metabolic inhibition, and liver damage [10]. Acetaminophen is detected in various ecosystems due to imperfect metabolism and mineralization that cause a severe damage to the human health [3]. Thus, the elimination from the aquaculture is a must to satisfy the environmental standards.

Presently, the need of engineers in the worldwide is to reach to sustainable wastewater treatments over the conventional technologies [11]. In the view of such concept, contaminated water recovery and waste disposal and management is essential for the reclamation pathway science for sustainability [12]. The conventional treatment methods introduced are not effective due to they are leading to incomplete and insufficient mineralization and removals or and the presence of unavoidable issues including the secondary wastes or using expensive chemicals that makes such processes are costive. “*Green*” emerging pollutants feature of treatment is an essential in the modern world to satisfy the sustainability trend [13, 14]. Physical adsorption is categorized as on of the inexpensive methods especially when it is based on the use of waste substances to overcome the process cost, accessibility and real applications. Numerous materials have been applied as wastewater adsorbents. However, according to the authors’ knowledge, augmentation of cellulosic type derived waste with magnetic material, as acetaminophen adsorbent is not applied so far [15]. Such reinforcement can posses the merit of recoverable adsorbent to be reused for successive treatments. Also, the application of the nanoscale adsorbent in pharmaceuticals contaminated streams elimination has recently attained the scientists’ attention since their effective particle size that leading to an efficient treatment.

**Fig 1:**
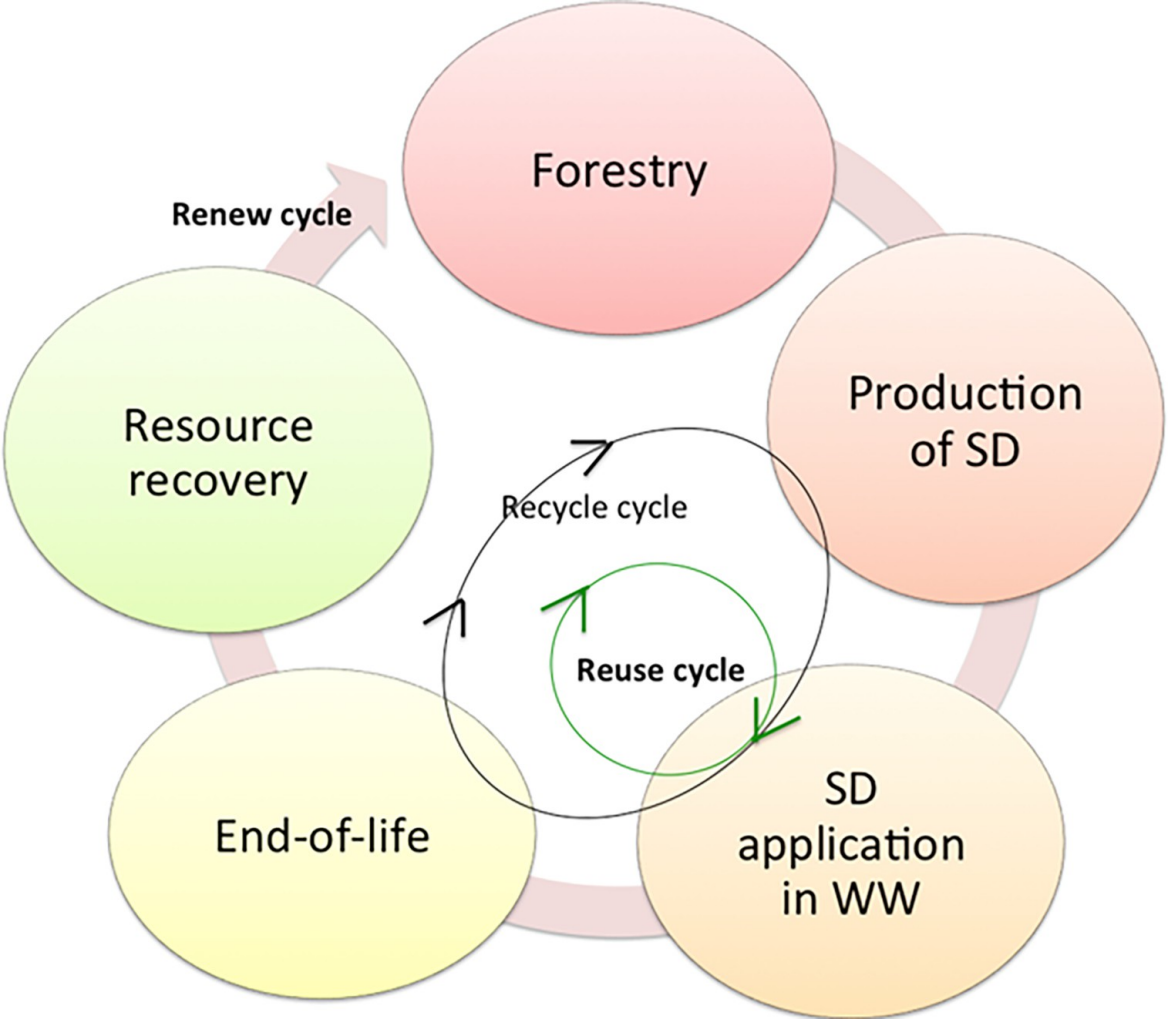
Circular economy approach of sawdust material.

Sawdust (SD) that is signified as natural source of cellulose fiber. Such cellulosic fiber could be considered as inexpensive, effective, extreme water adsorption and its great ability for chemical modification using metal oxides to be a nanocomposite [16, 17]. Cellulosic fiber is widely applied as adsorbent substance due to their surface affinity for adsorption as a sustainable waste material (see Fig 1). Agro-cellulosic waste is attained from wood processing. It is found the use of such waste in the field of wastewater treatment could limit such waste and converting it to a value added material via reuse cycle. Also, when it is recycled after wastewater treatment as acyclic uses it converting it to end-of-life waste. Thus, the recover, reuse and recycle are essential.

In this regard, the work investigated herein is keen to demesne of sustainable win-win remediation via converting cellulosic *Beachwood* sawdust fibres waste into a reliable magnetized adsorbent substance through a quick, simple and solventless methodology for microwave assisted composite preparation as a novel nanocellulosic magnetic composite based on magnetite and modified sawdust by product. But, its application in augmentation with nanoparticles for the pharmaceutical adsorbent is still limited [18–20]. According to this concept, further research is essential to reach to the practical applications. Currently, hybrid substance as a non-toxic and environmentally benign hybrid material could be applied for acetaminophen adsorption [21–23]. The composite might form a stable complexion with metal oxide that is then dragged away from the water and removed by settling facility.

In the realm of environmental sustainability concept, converting sawdust into cellulosic material augmented with magnetite is converting it to a sustainable catalyst that could be recovered easily and introduced for use in wastewater treatment for successive cycles. Thus, the magnetic adsorbent could be easily recovered for sustainable use. The main goal of this investigation is to investigate the feasibility of conjugation of metal oxide with cellulosic waste to be an effective sustainable adsorbent substance. X-ray diffraction (XRD), transmission electron microscope (TEM) and Vibrating sample magnetometer (VSM) are used to assess the structure and morphology of the substance as well as its magnetic affinity. Different process parameters are investigated and the thermodynamics is and kinetic modeling are also assess for introducing its real potential application.

### 1.1. Bibliometric tracking of the sawdust investigations trends

**Fig 2:**
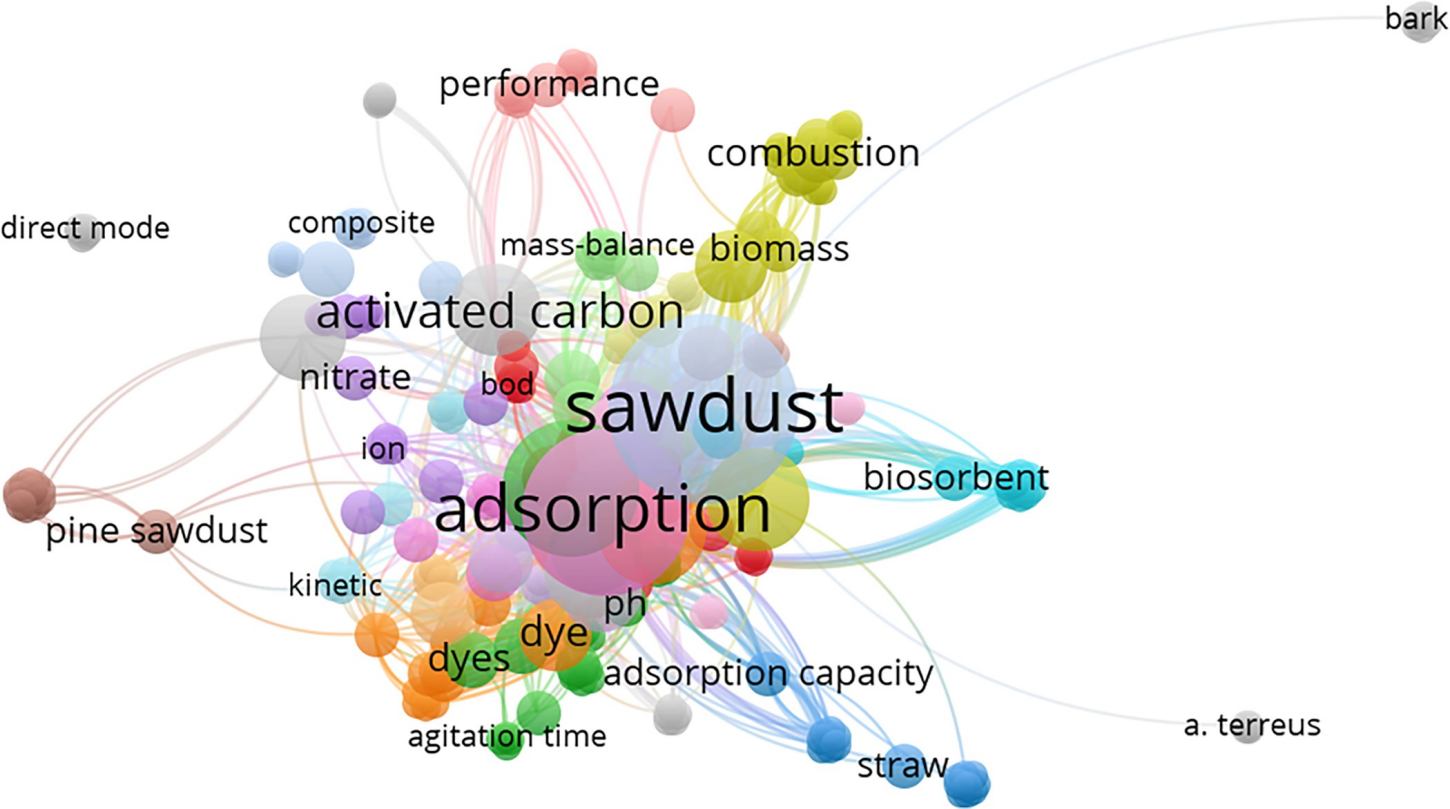
Bibliometric analysis mapping over the previous two decades based on overlay density image based on sawdust in wastewater treatment.

Bibliometric tracking technique is applied as a valued tool for appropriate studies cited analysis in particular subject. In such analysis a link may be key aspects of a certain topic [24]. Bibliometric mapping is attained to signify the supreme cited papers that are available in the articles database and the examination of relations concerning the terms achieved. Therefore, an examination of the “*Web of Science*” database by the words of “sawdust AND wastewater treatment” was conducted in scientific journals through the years in the duration of 2000–2021 with an increased number in the research articles conducted in the topic that help in further development of wastewater treatment to attain sustainability through cradle-to-cradle (CRD) ecology. The profile of the current research work conducted in the topic of wastewater and sawdust applications are given in Fig 2. The bibliometric network mapping created via VOSviewer (version 1.6.16.0) analyzing the keyword occurrence and the clusters represent overlap imagining of the bibliometric representation of hotspots displayed in Fig 2. The magnitude of clusters is associated to the study significance and implication in the field. As seen from the figure, such clusters in the visualized mapping scheme, a special attention could be paid for the adsorption technique for the sawdust material. Thus, it could be addressed that it is not used in the field of acetaminophen adsorption as a composed sawdust material.

### 1.2. Pharmaceutical contaminated wastewater treatment with low-cost adsorbents

Currently, worldwide is suffering from the numerous kinds of substances that disposed from pharmaceutical industrial discharge which are signified as organics and inorganics materials. Those substances are highly toxic since they are carcinogenic in nature. Such discharge when leaking to the ecosystem causing a massive damage the quality of water supply. Through the twenty-century, various low-cost adsorbent substances have been attaining the scientists’ attention and suggested and introduced as favorable sorbent materials for eradicating numerous contaminants from wastewater effluent. According to the data that is previously cited in the research articles, common pharmaceuticals that are extensively eliminated via the use of low-cost adsorbents are designated in details.

One of today’s developing industrial sector, pharmaceuticals are signified as one of the emerging pollutants. But, such release onto the ecosystem is a concern is a danger since they are low biodegradable, signified as high persist and bio-accumulate. Such materials have varied groups including antibiotics, blood-lipid controllers, anti-inflammatory agents and steroidal hormones. Numerous approaches have been applied to eliminate such substances from aqueous effluent such as advanced oxidation techniques, physical flotation, electrochemical technologies and biological techniques. Nevertheless, such procedures are limited since their high operational or maintenance costs or generating secondary more toxic pollutants. Application of adsorption treatments for those contaminants (Table 1) is a promising technique due to its diverse merits including their simple and manageable operation, cost efficient materials and high efficacy in treatments and elimination of pollutants and no further secondary toxic intermediates or by products formation.

**Table 1:** Pharmaceuticals elimination through various cost-effective substances.

Adsorbent	Pollutant	Isotherm time	pH	Temperature	Adsorbent dose	Pollutant concentration	Adsorption capacity	Ref.
Magnetite: sawdust	Acetaminophen	2 h	7.0	32°C	2.0 g/L	200 mg/L	7.1 mg/g	This work
Carbons material	Tetracycline	--	7.0	25°C	1.0 g	60 mg/L	910 mg/g	[25]
Charcoal from Bamboo	Tetracycline	24 h	7.0	25°C	-	100 mg/L	46.5 mg/g	[26]
Activated carbon	Acitaminophen	6 h	3.0	30°C	6 mg	120 mg/L	218 mg/g	[28]
Activated carbon	Clofibric acid	6 h	3.0	30°C	6 mg	120 mg/L	113 mg/g	[28]
Coffee waste/magnetite	Tetracycline	120 min	5.0	55°C	1.0 g	200 mg/L	286 mg/g	[25]
Olive stones carbon	Acitaminophen	-		25°C		10 mg/L	89mg/g	[30]
Palygorskite clay	Amitriptyline	-	6.0	-	-	-	0.168 mmol/g	[31]
Tea bags waste	Sulfamethazine	pH 3.0	3.0	-	-	-	34 mg/g	[32]
Treated Industrial cork	Ibuprofen	17 h	-	-	6 mg	150 mg/L	175 mg/g	[33]
Coffee and almond shell wastes	Datrizoate	50 min	7.0	25°C	-	100 mg/L	0.28 mmol/g	[34]
Coffee and almond shell wastes	Metronidazole	50 min	7.0	25°C	-	100 mg/L	0.13 mmol/g	[34]
Coffee and almond shell wastes	Dimetridazole	50 min	7.0	25°C	-	100 mg/L	0.89 mmol/g	[34]

According to the data tabulated in Table 1, numerous trails have been introduced that applying low-cost adsorbent materials for various pharmaceuticals removals from aqueous effluent. Oladipo and his co-workers [25] concluded the viability of applying coffee residue/magnetite as an adsorbent material. Whereas, Hubetska et al. [26] checked the feasibility of mesoporous carbons derived from sucrose and polystyrene for wastewater treatment. Additionally, Liao et al. [27] investigated the possibility of applying bamboo charcoal for adsorbing tetracycline in aqueous effluent. Such results reported that the applied low-cost adsorbents could reach to the adsorption capacity reached to 910 mg/g. Marques and his co-workers [28] concluded efforts to apply activated carbon to eliminate the clofibric acid. Their results reported that the adsorption uptake could reach to 113 mg/g and thus their results is a promising to substitute the low-cost material with the high-cost substances. Also, ibuprofen removals is suggested by Mestre et al. [29] using low-cost materials with capacity of uptake reached to 175 mg/g. García-Mateos et al. [30] assessed the use of carbon originated from olive stones for acetaminophen removals. Also, the current study revealed an adsorption uptake at the room temperature with neutral pH and the adsorption capacity of 7.1 mg/g. Although, the adsorption capacity from the current work is a quite reasonable, it is essential to note that it is derived from a waste stream. It is noteworthy to mention that critical operating parameters could affect the capacity of adsorption uptake including operating pH value, initial adsorbate concentration, adsorbent dose. Commonly, the application of low-cost adsorbent substances is conveying such substances from wastes streams into value added materials for meeting the product merits of ecologically approachability and cost efficacy for numerous commercial and manufacturing applications.

### 1.3. Comparing different sawdust systems for wastewater treatments

The adsorption capacity of various pollutants over a variety of sawdust adsorbents is compared. Various sawdust-based technologies in the previous literature are reported and the data is tabulated in Table 2. It is notably to state that some suggestive systems are based on the augmentation of magnetite with sawdust, which introduces the recoverable adsorbents since the availability of magnetite converting the sawdust material to be a magnetized material [33]. The data revealed that the systems could be reached to almost complete removals that are 98% removal. Thus, the material performance in the current study showed superior pollutant removals. Also, it is investigated that the systems could remove various pollutants including heavy metals, emerging pollutants and heavy metals. The data summarized in Table 2 illustrates that the removals is ranged from 5 to 98% due to the type of pollutant removed. Furthermore, the operating condition including the adsorbent nature is also posses a crucial role in the adsorption uptake. For instance, some of the heavy metal removals showed higher adoption uptake through the removal efficiency [9]. Also the operating conditions is in diverse range including contact time, pH and adsorbent dose, which are linked to both the type of the adsorbent and the adsorbate. On the other hand, it might be argued that the presence of various functional groups depending on the nature of the materials also affects the adsorption process [11].

**Table 2:** Various sawdust-based adsorbents for different wastewater types.

Type of sawdust adsorbent	Adsorbent dose	Pollutant	pH	T/°C	Isotherm time, min	Removal/ %	Ref.
Magnetite: sawdust	2.0 g/L	Acetaminophen	7.0	32°C	2 h	83	This work
sawdust		Acid Blue 25 dye	4.0	26°C	2 h	59	[25]
Neem sawdust		Basic Crystal violet dye	7.2	NA	-	5	[26]
Neem sawdust		Basic Malachite Green dye	7.2	31°C	-	85	[26]
cellulose nanocrystal/ sawdust	0.1 g	Vanadium	3.0	45°C	4 h	95	[27]
Sawdust/Magnetite	0.2 g	Ca(II)	3.0	-	50 min	98	[9]
cellulose nanocrystal/ sawdust		Cu(II) and Pb(II)	7.0	23°C	6 h	90	[28]
ZrO_2_- sawdust		Arsenic	7.0	28°C	24 h	50	[11]
cellulose nanocrystal /ZnO	0.1 g	methylene blue	10.0	45° C	6.5 h	90	[7]
magnetic sawdust carbon @magnetite/ sawdust		methylene blue	7.0	55° C	30 min	85	[29]
sawdust	2.0 g	Lead	6.0	NA	50 min	98	[30]
magnetite/ sawdust		strontium	6.74	NA	30 min	25	[31]
Zerovalent Iron / sawdust		Arsenic (III)	7.74	NA	4 h	13	[32]
ZnO, sawdust & ZnOnp- sawdust		Pb (II)	8.0	20° C	100 min	70	[33]

Furthermore, it is reported that in some cases a minimal isotherm time is required which also reduces the operating cost of such system when it is converted to a real scale application. However, it is essential to mention that adsorption treatment technology might result in the formation of secondary wastes, which is essential to be treated. But, the use of recoverable catalyst could help in converting the materials to be recoverable [25, 33]. Further, the waste material when sawdust is available as adsorbent might convert the waste after treatment into a fuel-based effluent which valorizing their applications.

## 2. Experimental section

### 2.1. Materials

*Beachwood* sawdust substance was collected from a local carpenter and wood-processing workshops in Menoufia Governorate, Egypt. H_2_O_2_ (3%, w/w) and HCl (2.5 N) were used for chemical hydrolysis and bleaching of sawdust through treatments, respectively. Ferrous and Ferric Sulfate (delivered by Qualikems Chemicals, India) were used as the pre-coursers to prepare magnetite nanoparticles. H_2_O_2_ (40%, w/w) is added to initiate the oxidation system. pH adjusted using diluted H_2_SO_4_ and NaOH. All chemicals are used as delivered without extra purification.

### 2.2. Synthesis of composite fibers

Initially, the collected sawdust, SD was washed several times with distilled water (DW) before it exposed to over night oven drying (105°C). Subsequently, the cellulosic fibres are isolated from the clean dried SD waste through hydrolysis. In such technique, 25 g SD was subjected to 200 ml of (2.5 N) HCl at 90°C for 15 minutes of reaction time. Afterwards, the mixture was filtered and then washed repetitively using DW till pH 7.0 is attained. The cellulose fibres are attained and exposed to oven drying (60°C) till constant weight is obtained. The attained material was crushed to obtain a fine powder through subjection into milling by a ball mill machine (300 rpm for a period of 10 hours each hr). Bleaching process is then conducted over the cellulose powder via using H_2_O_2_ (3%) and the solution is heated for 1 h (90°C) during stirring. Finally, the mixture was filtered and rinsed with DW till pH 7.0 and the obtainable cellulosic fibres were oven dried (105°C) till constant weight is achieved. The isolated SD material is kept in a tightly closed polyethylene bottles.

Environmentally benign co-precipitation route was applied to prepare the magnetite, Fe_3_O_4_ nanoparticles using 2 and 1 mol of ferrous- and ferric sulphate, respectively as precursors that are hybrid addition through their specific amounts with DW. Furthermore, in order to precipitate the nanoparticles, NaOH solution was added in drops till the solution pH is recorded 11 to reach to their isoelectric point [15]. Then, the precipitate is attained in the solution and the mixture was subsequently exposed to a constant mixing (at 80°C). Subsequently, the as-prepared nanoparticles were subjected to repeatedly washing using DW till the neutral pH is reached to remove any remaining Na_2_SO_4_ nor NaOH in the solution. Then, the attained solution was left for settling prior to filtration and washed with DW prior to oven drying (60°C) [35].

**Fig 3:**
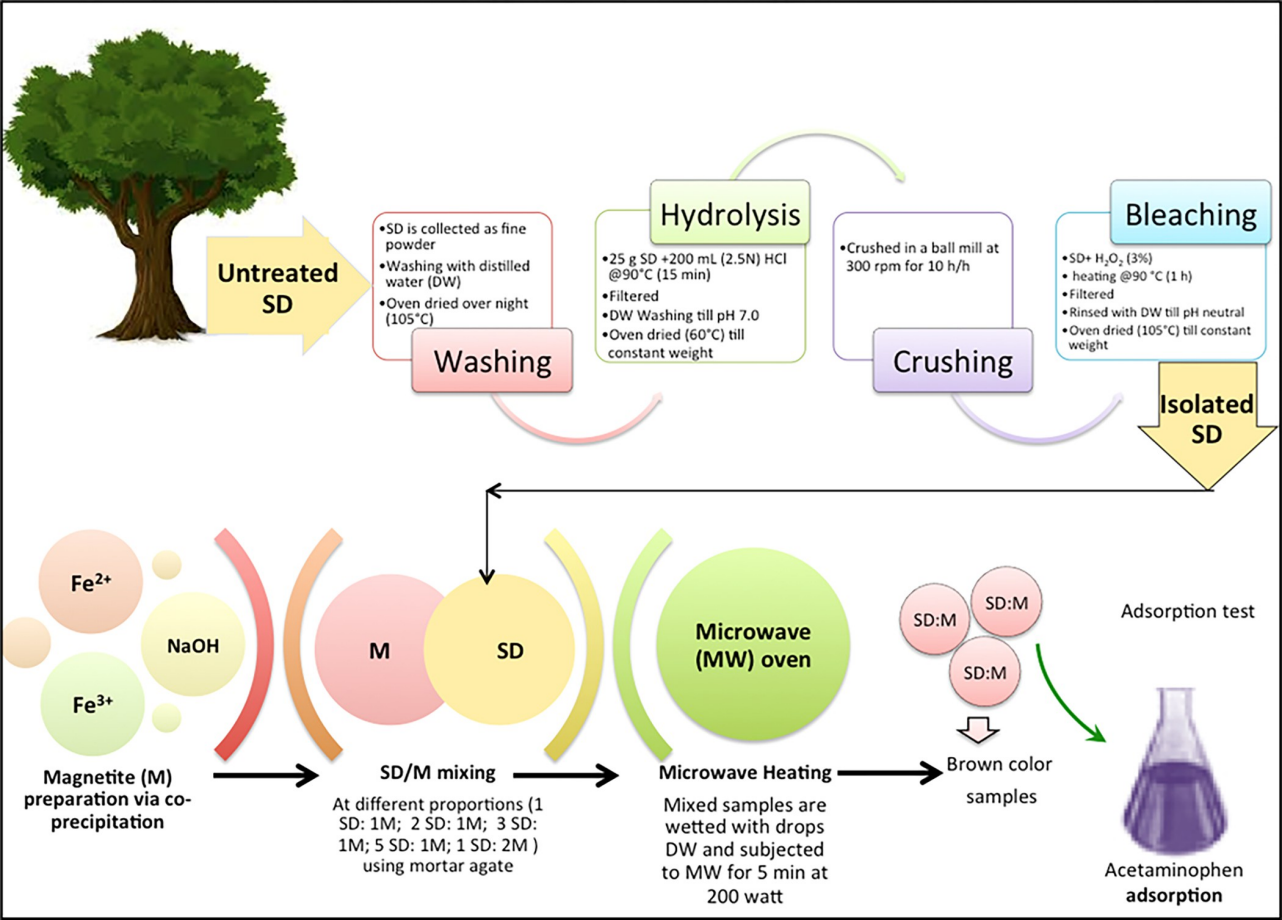
Schematic representation of M:SD preparation steps to be an adsorbent.

The produced magnetite Fe_3_O_4_ nanoparticles that is labeled as (M) were mixed with isolated SD in various mass ratio proportions of M:SD (1:1, 1:2, 1:3, 1:5 and 2:1). Thereafter, the samples are carefully grinded by mortar of agate. The solid mixture samples are preserved in a small glass container of petri dish and wetted with few drops of DW before subjected to household microwave oven for 5 minutes at a power of 200 watts according to the procedure described elsewhere. The attained M:SD nanocomposite looks homogeneous brownish in colour. The detailed schematic steps of preparing the adsorbent composite materials are presented in graphical illustration of Fig 3.

### 2.3. Adsorption procedure

A stock solution of 1000-mg/L of acetaminophen drug was initially prepared, and a dilution was carried out to attain the essential concentrations that are ranged from 20 to 200 mg/L. A volume of 10 mL of acetaminophen aliquot samples was poured into a glass sealed vessels. Initially, the pH of the aqueous mixture is attuned, when needed, prior to the nanoparticle’s addition. Solutions of NaOH of 1 N and diluted H_2_SO_4_ were applied to attain the required pH of the aqueous solution. The adsorbent dose is checked in the range of 0.25 to 6.0 g/L. After the nanoparticles addition to act as a nanofluid adsorbent material, the aqueous mixture and the reagent are subjected to magnetic stirring to get a good contact between the adsorbent and the pollutant. Consequently, after the definite isotherm time is investigated, the samples were exposed for spectrophotometric analysis and the adsorbent material is subjected for filtration removal through a syringe micro filter. Following the contact time investigation, all the adsorption parameters including pH influence, temperature effect, acetaminophen loading and adsorbent dose are all checked. All the analysis was shown in three triplicates, and the average values are monitored. Then the recorded data, even in Tables or Figures, are the average values of at least three as an adsorption capacity (mg/g) according to the relation ($\left(q_{e}=(C_{o}-C_{i})\frac{V}{m}\right)$), where C_o_ and C_t_ are respectively initial and final acetaminophen concentration, V is the solution volume in liter and m is mass of sawdust magnetite adsorbent material used (g).

### 2.4. Characterization

The microstructure, morphology and composition of the synthesized magnetite, SD and M:SD samples are characterized. The structure of the arranged material composite was analyzed by X-radiation powder diffractometry XR Phillips X’pert (MPD3040) diffractometer with Cu-K_α_ radiation at ambient conditions (λ = 1.5406 Å). This investigation used step-scan mode with intensities over the 2θ range of 5–80°. Moreover, the morphologies of the prepared magnetite, SD as well as M:SD samples, were examined and pictured through SEM (Field-emission scanning electron microscope with a model is Quanta FEG 250) with different magnifications (x8000 and x60000) and Transmission Electron Microscope (TEM) of the model type Tecnai G20, FEI with applied typical magnifications of x8000 and x60000. SEM was supplemented by energy-dispersive X-ray spectroscopy (EDS). The main contained metal oxides in the SD and M:SD (1:3) were examined through the energy dispersive spectrum. Moreover, the magnetic characteristics were highlighted and investigated by measuring the vibration sample magnetometer (VSM, Lake Shore Cryotronics, Model 7410) at room temperature.

## 3. Results and discussions

### 3.1. Characterization of nanocomposite

#### 3.1.1. XRD analysis

**Fig 4:**
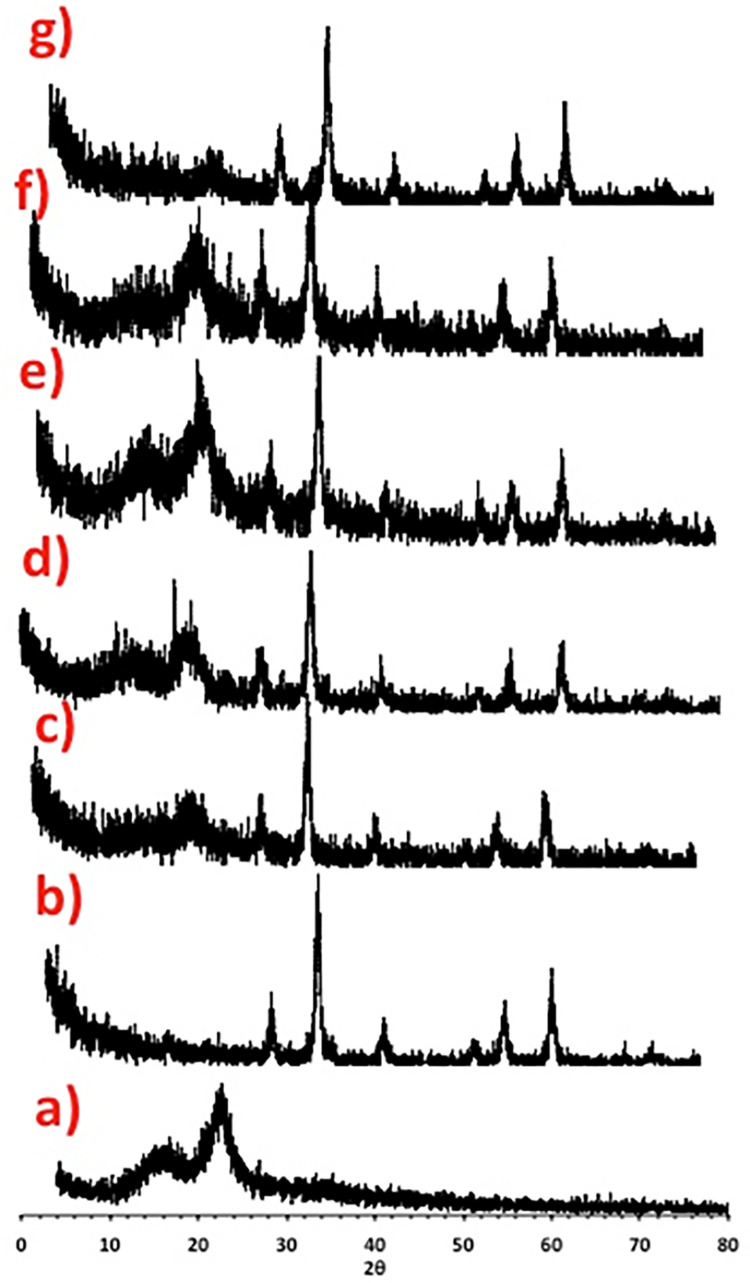
X-ray diffraction of different adsorbents: (a) M, (b) SD, (c) M:SD (1:1), (d) M:SD (1:2), (e) M:SD (1:3), (f) M:SD (1:5) and (g) M:SD (2:1).

For the object of examining the crystalline behavior of the prepared samples, XRD analysis was applied and the data of the prepared M:SD composite with compared with the pristine material. The data displayed in Fig 4 shows the distinct peaks of pristine magnetite (Fig 4A), SD (Fig 4B), M:SD (1:1) (Fig 4C), M:SD (1:2) (Fig 4D), M:SD (1:3) (Fig 4E), M:SD (1:5) (Fig 4F) and M:SD (2:1) (Fig 4G). As displayed in Fig 3, two distinct peaks were detected in pristine SD at diffraction angles (2θ) of 15.6° and 22.4° that verifies the presence of lattice plans (101) and (002) of cellulose I. Additionally, a tiny extra peak was noted at 2θ of 34.7°, corresponding to the (400) phase of crystalline cellulose I. Furthermore, the attained peaks corresponding to the investigated substance sample exposed the ferrite substance’s single face-cantered cubic (FCC) spinel structure. The most intensive peaks (311), (440), and (220) which are corresponding to 2θ of 35.52, 62.84 and 30.2°, respectively [15]. Moreover, the presence of other crystal plans, which is also associated with the presence of magnetite, such as (111), (422), (511), and (533) that are corresponding to 17, 54.5, 66.7, and 74.8°, respectively which are attributed to the presence of magnetite exploring the successful creation of Fe_3_O_4_. Similar peaks were attained for the various M:SD samples, implying the successful augmentation of Fe_3_O_4_ with sawdust, which confirms the presence of magnetite peaks in SD.

#### 3.1.2. SEM images and EDX analysis

**Fig 5:**
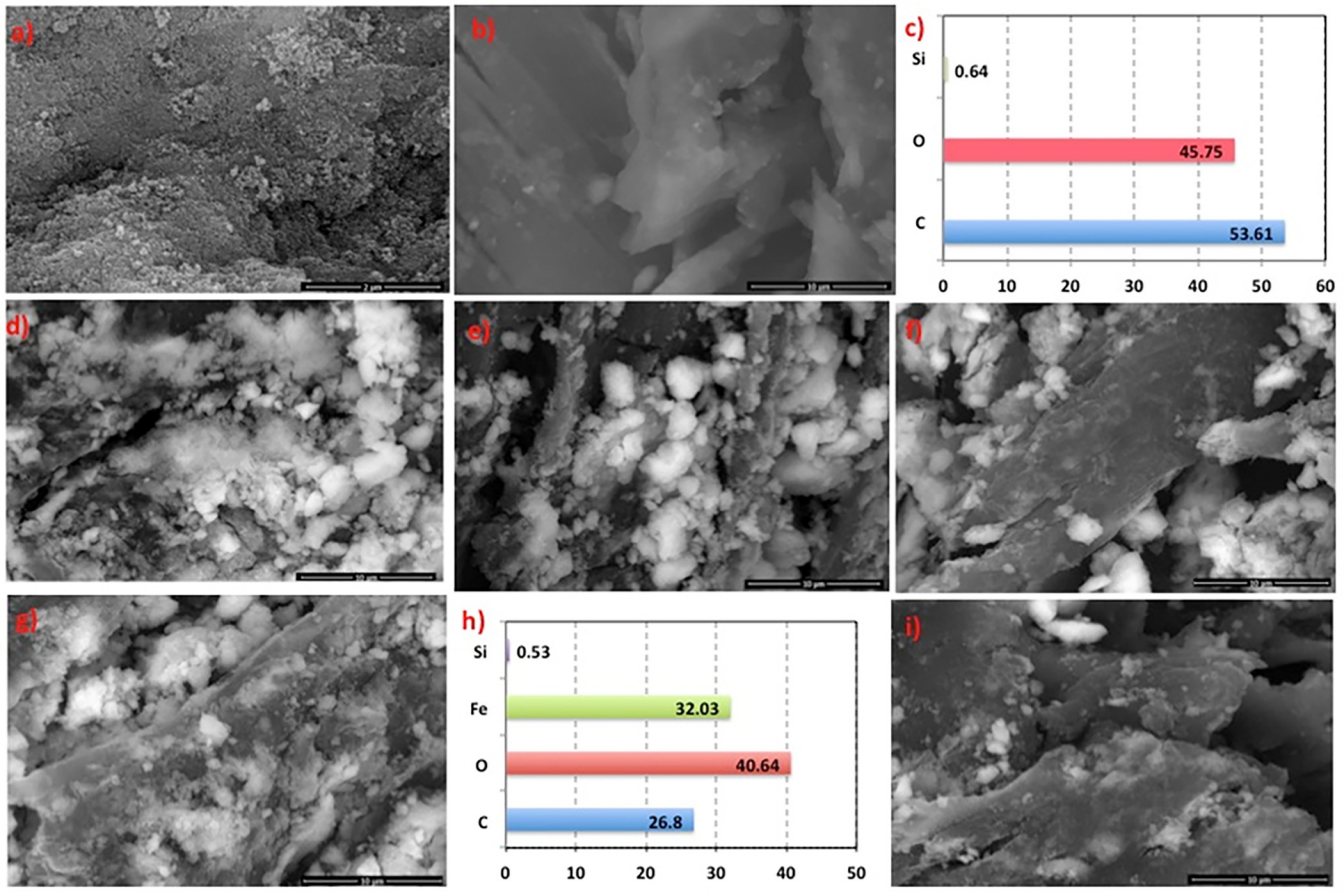
SEM micrographs and EDX images of a) pristine SEM M, b) pristine SD SEM, c) SEM and d) EDX of M:SD (1:1), e) SEM of M:SD (1:2), f) SEM M:SD (1:3), g) SEM and h) EDX M:SD (1:5) and i) SEM M:SD (2:1).

For the object of exploring the surface morphology of the pristine treated sawdust and the composite nanoparticles, SEM images were investigated (Fig 5). The size of the pristine M (Fig 5A) and SD (Fig 5B) was in hundreds of microns and it controlled a structure of layers observance with various arranged vessels between the layers. Such vessels and layers provide considerable space to adsorb pollutants (acetaminophen) in aqueous effluent. The sawdust before augmentation with magnetite, the surface of vessel was smooth, and there were several folds between each vessel. However, magnetite aggregates in a random distribution on the SD surface after magnetic modification, as illustrated in Fig 5. For SD supplemented with magnetite (Fig 5(C)), the gloss change of the surface was due to the cover of magnetite for the SD surface. Also, the layer of SD that may inhibit them from being oxidized and detached uniformly covered the magnetite combinations. The SEM analysis illustrates that the percentage of SD added changes the intrinsic structure of M: SD due to the increased or decreased mass-weight ratio of M and SD, indicating the successful mixing of magnetite as seen in images of (Fig 5C, 5E–5G and 5I).

The EDX results of pristine SD (Fig 5C) and SD augmented with Fe_3_O_4_ (M:SD-1:3) (Fig 5H) nanoparticles composite are exhibited. The surface of solo SD was principally consisting of C (53.61%), Si (0.64%) and O (45.75%), while SD augmented with magnetite nanoparticles was mainly comprised of C (26.63%), O (40.54%), Si (0.51%) and Fe (32.22%). The C percent presented a decline after adjustment and modifications, from 53.61% to 26.63%, due to SD, mainly consisting of C and O. In the spectrum of SD supplemented with magnetite nanoparticles, the percent of Fe appeared, indicating the successful mixing with magnetite and SD. The data confirms the uniform atom distribution, equivalent well with the SEM image.

#### 3.1.3. TEM images

**Fig 6:**
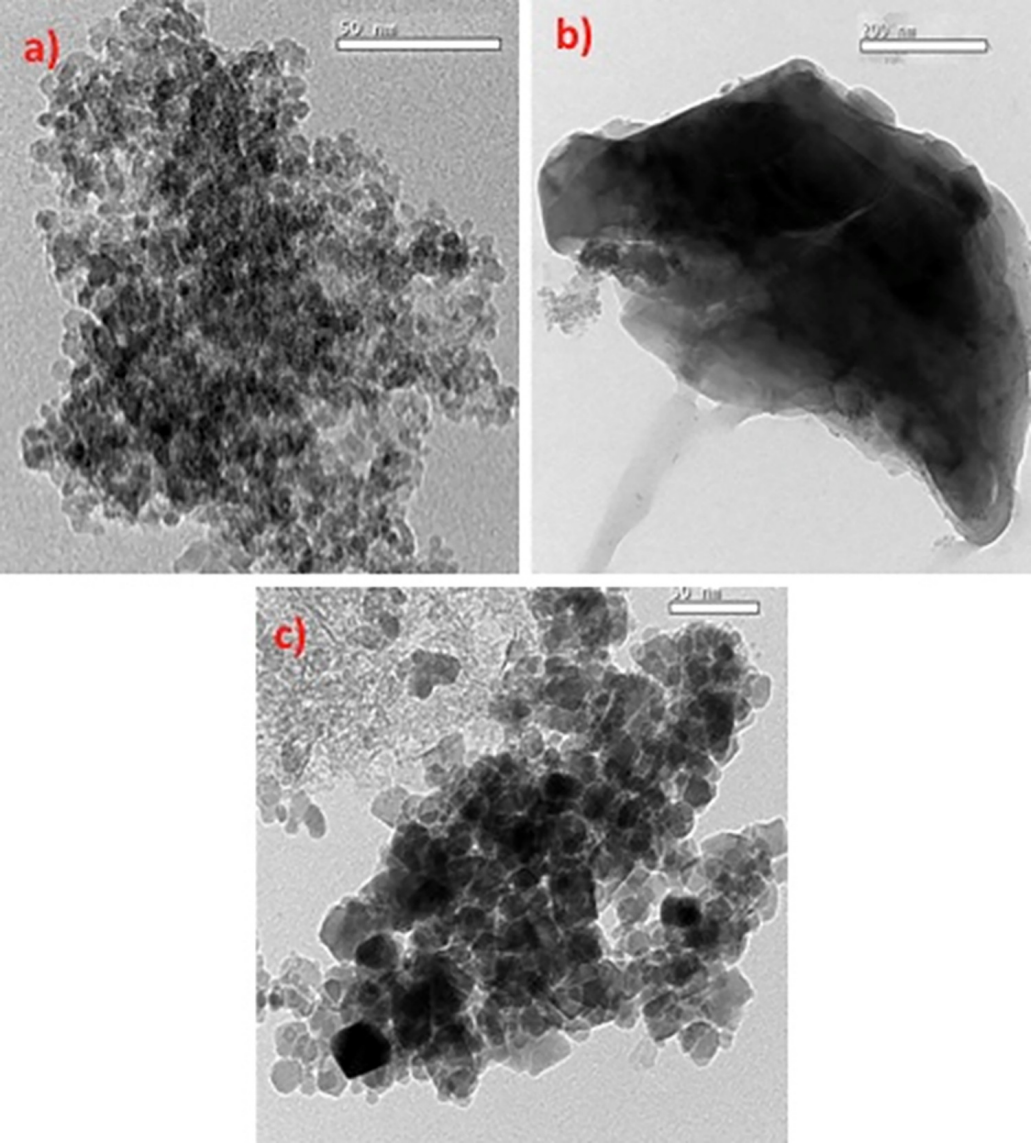
TEM micrographs of a) pristine M, b) pristine SD and c) composite of M:SD (2:1).

The magnetite nanoparticles, isolated cellulosic *Beachwood* sawdust fibres and *Beachwood* sawdust fibres augmented magnetite nanoparticles as a composite substance are explored under transmission micro-scope (Fig 6). Fig 6(B) explores that the TEM micrograph of the prepared pristine magnetite nanoparticles, the particles are nearly spherical in shape. Image of Fig 6(B) signified a sheet-like morphology for the cellulosic sawdust material. Fig 6(C) depicted the sheet-like morphology of the sawdust that is illustrated to be uniformly covered with dense aggregates spherical of magnetite nanoparticles over the sheet *Beachwood* sawdust surface.

The data of the specific surface area S_BET_ is assessed to investigate the characteristic of the adsorbent material. The data revealed that magnetite/sawdust composite possess a surface area of 78 m^2^/g.

#### 3.1.4. VSM analysis

**Fig 7:**
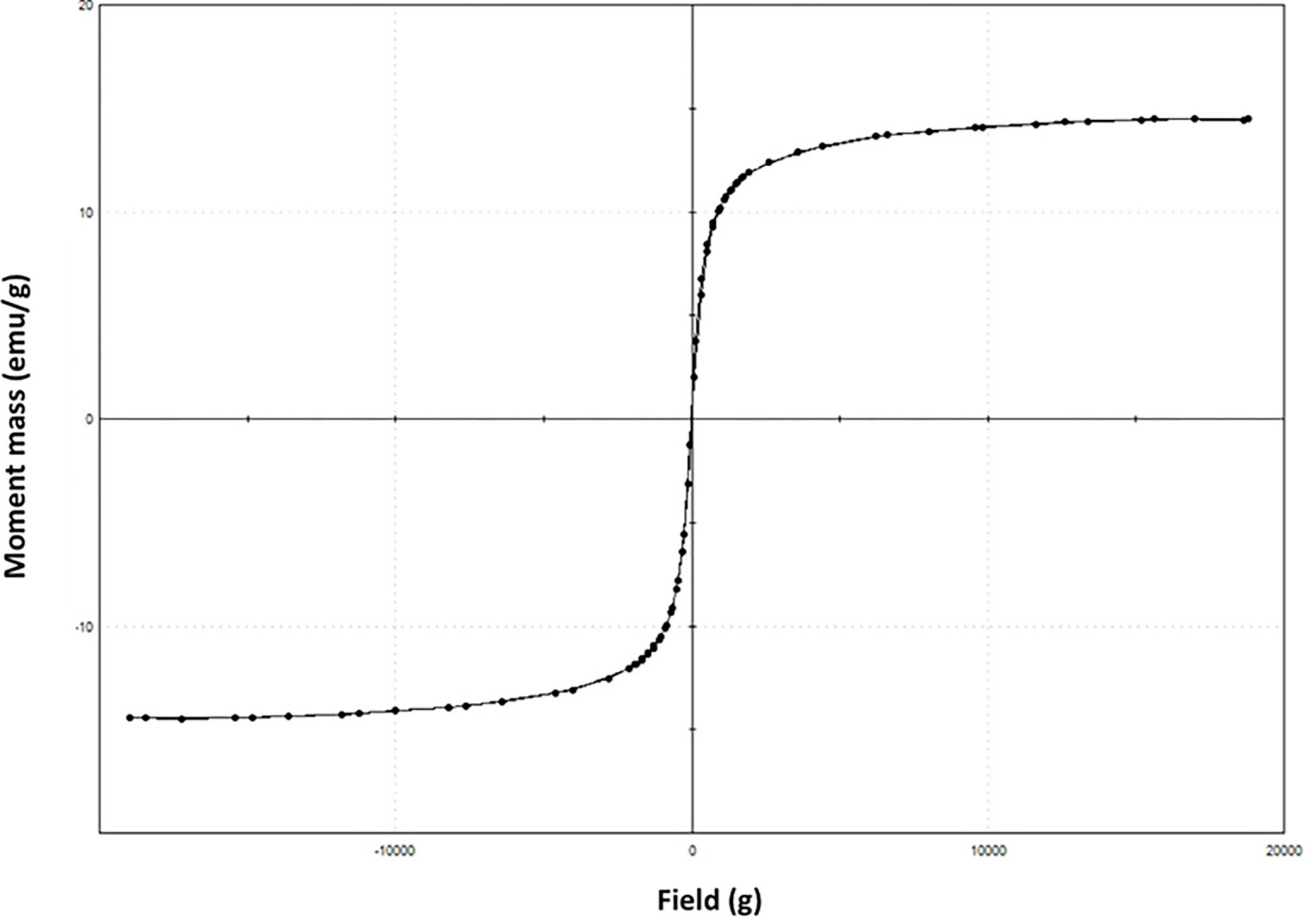
Magnetic properties through VSM of M:SD (2:1) composite.

Vibrating sample magnetometer (VSM) assessments the magnetic behavior of the sawdust/magnetite nanocomposite substance. Fig 7 exhibit magnetization loop for M:SD (2:1) nanoparticles at room temperature. The magnetic hysteresis curve displays the magnetic performance. The material exhibits a narrow hysteresis curve with a small value of coercivity and retentivity. VSM data reveals that the coercivity, Hci, that is the field needed to magnetize the prepared substance (18.077 G) and retentively, Mr, that is the field required to demagnetize the prepared substance (0.46203 emu/g). However, the saturation magnetization value of the magnetite nanoparticles was noted (14.483 emu/g) is a sign for above such value, the material could not be magnetized. Therefore, from the hysteresis loop results indicate that magnetic sawdust nanocomposite could be easily magnetize and demagnetize.

### 3.2. Studies on Synozol acetaminophen adsorption

#### 3.2.1. Acetaminophen adsorption time

**Fig 8:**
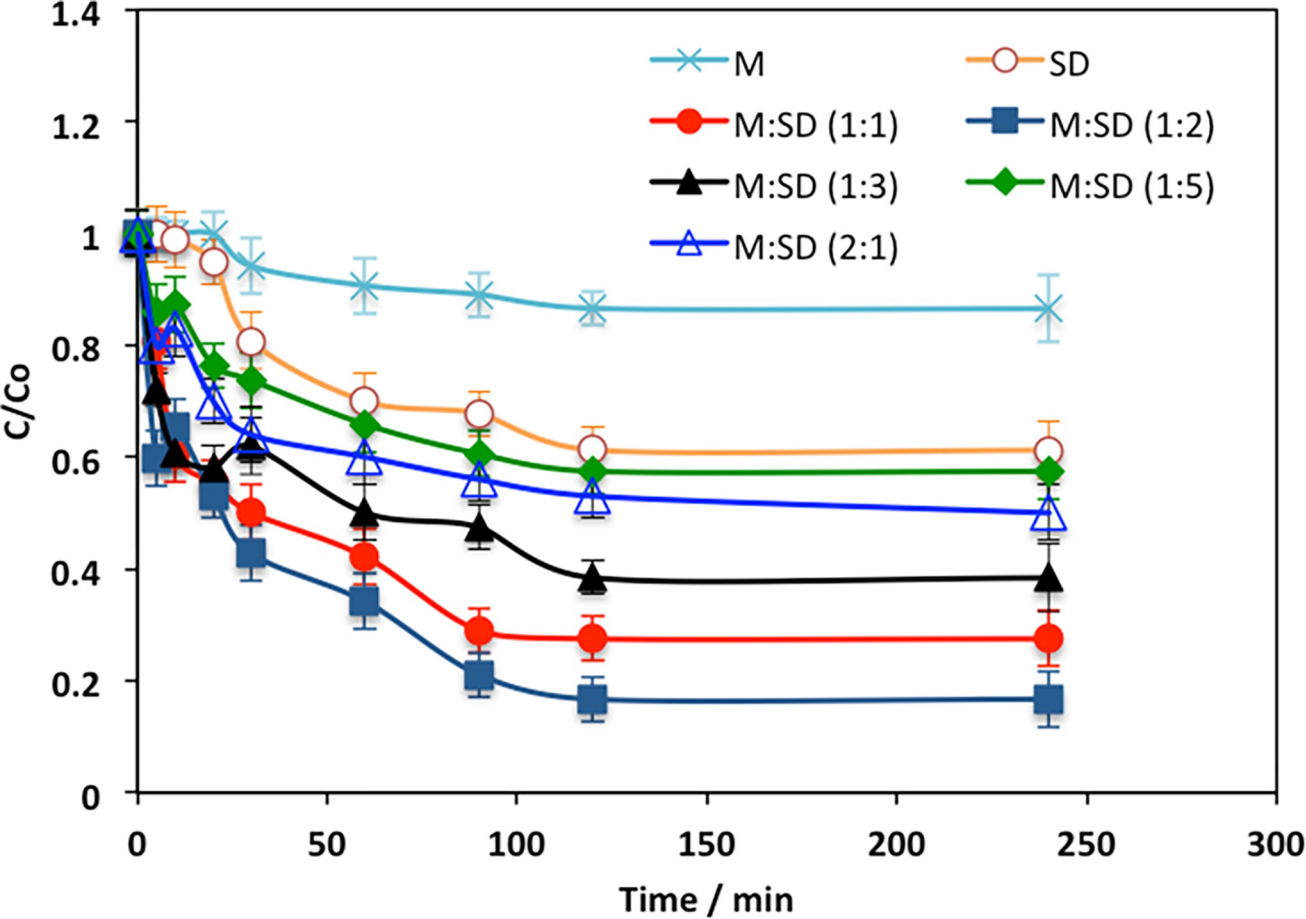
The influence of adsorption contact time on the uptake of acetaminophen was examined using various adsorbents based on sawdust.

Primarily, for the object of designing the adsorption system matrix, it is vital to assess the adsorption equilibrium adsorption time. The time-profile of acetaminophen sorption linked to using various adsorbents based on the sawdust/magnetite composite materials was evaluated at the ordinary room temperature. The data in Fig 8 displayed verifies that with the time processing the adsorption tendency presented as acetaminophen concentration reduction is achieved for all the adsorbents used. Overall, the acetaminophen adsorption capacity is the highest corresponding to the use of M:SD (1:2) composite. The majority of the acetaminophen sorbed within the initial 2 hours of contact time. This might be recognized according to the X-ray diffraction data is the presence of cellulose as well as magnetite in the sample compared to the pristine SD and magnetite sample. Also, it is essential to mention that; such substances in the composite are effective in acetaminophen adsorption because of the ion exchange characteristics.

Moreover, compared to the other adsorbents (composite based substances), the optimal presence of cellulose and magnetite revealed the highest tendency for the acetaminophen is directly verified as projected its significance in raising the adsorption uptake. The negative charge of the cellulose/magnetite framework makes it function as a cation exchanger. Consequently, water molecules can attach to the framework, allowing for the exchange of acetaminophen molecules. It is evident that, regardless of the substances used, the sorption of acetaminophen does not increase beyond two hours of contact time. This is because the active sites on the adsorbent materials become saturated and accumulated with acetaminophen molecules, leaving no additional capacity for further sorption. A similar investigation is observed by previous investigators [15] in treating emerging pollutants contaminated wastewater effluent.

#### 3.2.2. Effect of initial acetaminophen loading

**Fig 9:**
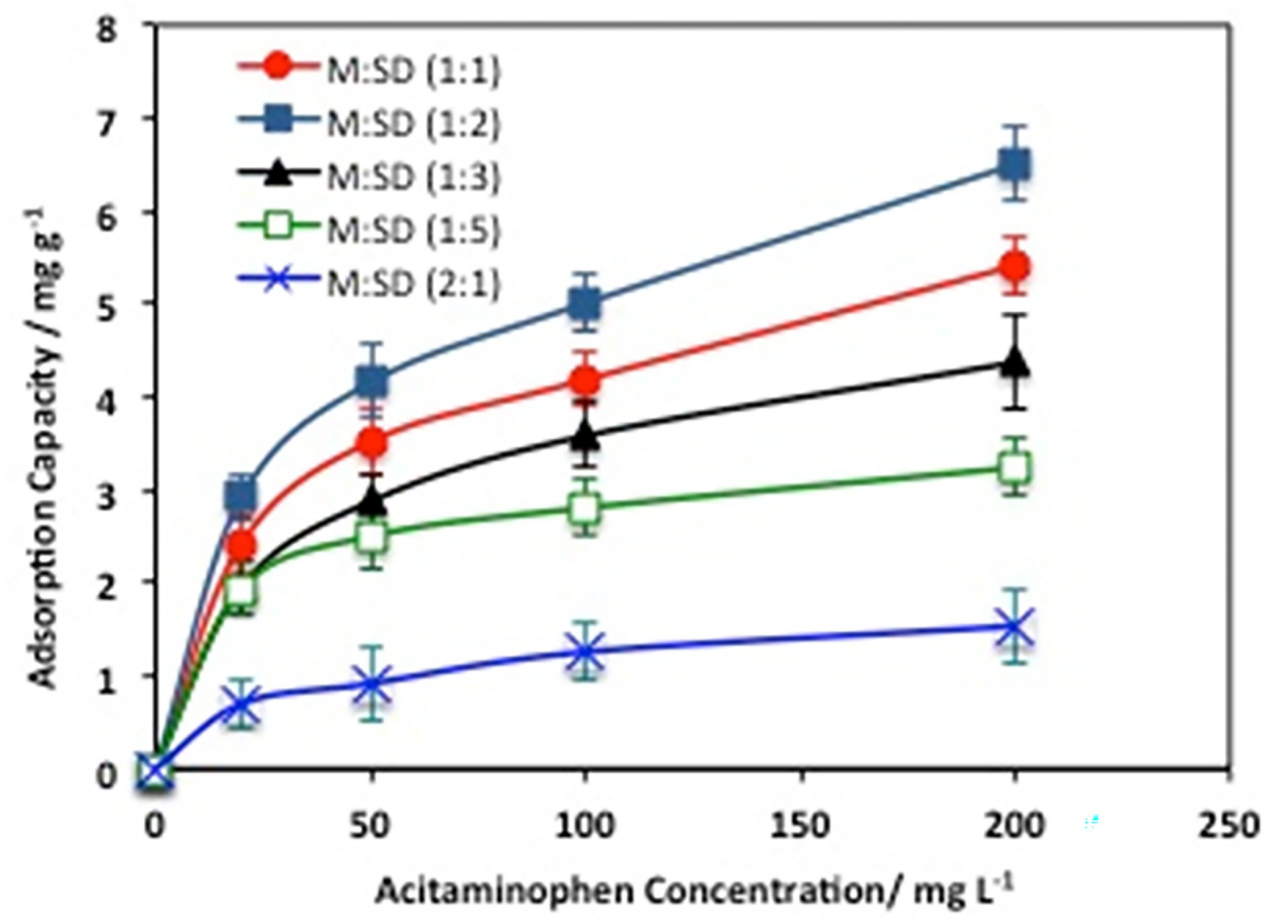
Effect of acetaminophen concentration on the adsorption capacity.

Fig 9(A) displays the effect of the initial acetaminophen concentration ranging from 20 to 200 mg/L at room temperature on adsorption capacity using the various sawdust/magnetite-based adsorbents. Overall, for all the applied materials, an obvious enhancement on the adsorption capacity with increasing the acetaminophen load due to the improvement interaction between the acetaminophen and M:SD materials at higher acetaminophen concentration. M:SD (1:2) displayed highest adsorption capacities overall, this might be attributed by the occurrence of cellulose and magnetite in optimal ratio for maximizing the adsorption uptake. Furthermore, The increased porosity and higher surface area of the sawdust enable the utilization of additional adsorption sites, thereby enhancing the overall adsorption capacity. The unit adsorption uptake of M:SD (1:2) elevated from 3.0 to 7.1 mg/g with the elevation of the acetaminophen loading in the aqueous media from 20 to 200 mg/L. This routine is linked to the concept of the increase in the initial acetaminophen concentration, excess acetaminophen molecules surrounded sawdust/magnetite sites and thereby improving the mass transfer rate between the adsorbent material and the solute adsorbed, the result is improvement in the adsorption capacity. Such results of improving the adsorption uptake with the increase in the adsorbate are in agreement with that previously reported in literature [25, 33].

Cellulose nanocrystal based bio-adsorbent augmented with magnetite posses’ great specific surface area with hydroxyl and anionic sulfate ester groups due to the presence of cellulosic material in addition to the presence of magnetite nanoparticles enlarge the composite surface area and enhancing the adsorption uptake. Thus, water molecules loaded with acetaminophen could be bonded onto the composite and then the acetaminophen molecules are exchangeable. Furthermore, the adsorption capacity differs according the various proportions of he blend. Thus, the extra sawdust in the composite might be associated to the active sites of sawdust and pores of cellulose such proportions in the blend might results in increasing the active sites for electrostatic interaction. This is verified by the increase in the cellulose in the sample, which increases the affinity of the adsorption efficiency. The presence of benzene rings in the acetaminophen structure results in a mechanism of π-π interaction that might also plays a crucial role with the adsorbent blend. Depending on the structure of the adsorbent and pollutant, due to the presence of nitrogen and hydrogen, formation of hydrogen bonds is possible in the structure. Thus, such functional groups might be affected with the amount of sawdust and magnetite in the sample [34–38].

#### 3.2.3. Acetaminophen adsorption related to operating parameters

**Fig 10:**
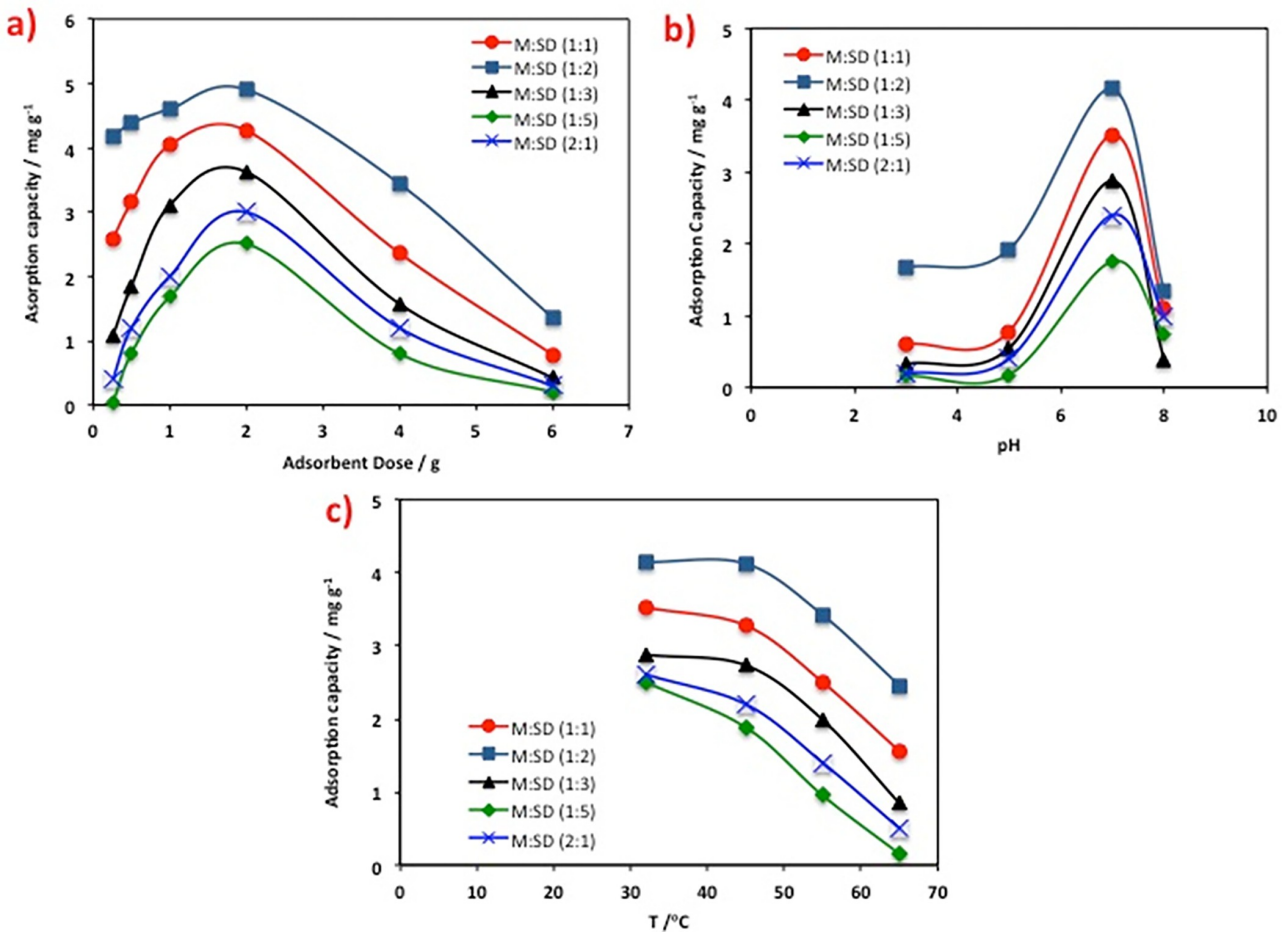
Effect of various adsorption parameters on the acetaminophen adsorption capacity (a) adsorbent dose, (b) pH and (c) on adsorption capacity of different saw dust/magnetite composite substances.

Acetaminophen uptake for the range of sawdust/magnetite composite based adsorbent dosages used (0.25 to 6 g/L), whereas all other operating parameters are kept fixed. Fig 10(A) shows that the adsorption capacity of acetaminophen improved with the dose of adsorbent increase up to 2.0 g/L. This could be associated with the increase in the available adsorption sites. But, the further increase in the adsorbent dosage the acetaminophen uptake is declined. The unsaturation of adsorption sites occurs as a result of the increased dosage of adsorbent while maintaining a constant concentration of adsorbate. Hence, at lower adsorbent dosages, acetaminophen adsorption is significantly reduced due to the inadequacy Also, the excess of the sawdust/magnetite composite might result in aggregation that means particulate interaction. This due to the adsorbent powder particles stick together and cause aggregation that decreases the active sites and adsorption capacity. of available sites [12]. This is thereby result in a reduction in a declined in the overall surface area and the diffusional path length is increased. Hence, the 2.0 g/L is recommended to be the optimal adsorbent dose. This exploration is in agreement with that previously reported by Tajernia et al. [10] in removing arsenic from wastewater.

For the practical application and real scale treatments, it is essential to study the outcome of the pH on the adsorption capacity. Numerous pH values of the aqueous effluent were evaluated varied from alkaline to acidic range while all other parameters are fixed. The data presented in Fig 10(B) showed that for all the adsorbents, the highest acetaminophen uptake is linked to the neutral pH value (7.0) that is preferred and is corresponding to the highest uptake. But, extra increase in the pH value to the alkaline range, the adsorption uptake is declined. The decrease in adsorption capacity at alkaline pH can be attributed to the absence of active sites in the sawdust/magnetite sorbent materials. Additionally, pH affects the surface charge of the composite. To verify such investigation, the point of zero charge (pH_PZC_) of the composite is used to characterize the composite adsorbent material since it indicates the pH of the material posses a net zero surface charge and all the active sites remain neutral. Thus, the solid addition method [36] is applied to evaluate the composite, which located the pH_PZC_ at 5.7. This confirms that the too acidic pH and the alkaline range are both not preferred for pollutant removal. The acidic medium results in excess in the amount of H+ that thereby competing the acetaminophen in adsorption on the composite surface. Also, the increase in the pH more than the point of zero charges the number of negatively charged sites on the surface of the substance upsurges and acetaminophen removal is further increase. This might be linked to the increase in the electrostatic attractions between the pollutant molecules and the adsorbent’s functional groups. However, the decrease in adsorption at high pH values for could be attributed to its partial hydrolysis [37].

Temperature influence on acetaminophen adsorption uptake was assessed. The reaction temperature was adjusted over the range of 32 to 65°C. The data displayed in Fig 10(C) demonstrated that acetaminophen sorption uptake and capacity declined with a temperature elevation. This might be demonstrated by the enhancement of the acetaminophen desorption stage in the adsorption process that verifies the process is exothermic in nature [5]. Moreover, the temperature elevation weakening the sorptive forces between the adsorption active sites on composite and the acetaminophen molecules. This also might be linked to the mobility of acetaminophen molecules are increases too. Hence, such increase in the mobility of molecules results in a decline in the contact and adsorption of acetaminophen by the suggested magnetite/sawdust adsorbent [38].

#### 3.2.4. Isotherm modeling

To assess the adsorption capacity of sawdust/magnetite substances, various isotherm models including Langmuir, Freundlich, Temkin, and Dubinin-Radushkevich (D-R) were examined. The Langmuir adsorption isotherm model suggests that the maximum adsorption capacity is associated with monolayer adsorption occurring on a homogeneous surface. (Eq. 1) [10]:

$$\frac{C_{e}}{q_{e}}=\frac{1}{K_{L}}+\frac{a_{L}}{K_{L}}C_{e}$$ (1)

$$Q_{o}=\frac{K_{L}}{a_{L}}$$ (2)

where *C*_*e*_ is the acetaminophen dose at equilibrium (mg/L), *q*_*e*_ is the equilibrium adsorption capacity, and *a*_*L*_ and *K*_*L*_ are Langmuir adsorption constants. *Q*_*o*_ is the monolayer adsorption capacity of the solid (mg/g). However, Freundlich model that describes the highly heterogeneous surface systems (Eq. 3), where it is described by the heterogeneity constant *1/n*, which signifies the favorability of the adsorption system.

$$\ln\left(q_{e}\right)=lnK_{F}+\frac{1}{n}lnC_{e}$$ (3)

where *K*_*F*_ is Freundlich constant that relate to the adsorption capacity of the solid (L/g) [25]. Furthermore, D-R isotherm is applied to evaluate the nature of the adsorption system since it accepts the adsorption is limited to monolayer coverage with no assumption for tendency of a homogeneous surface (Eqs. 4, 5). This isotherm model might be applied to assess the adsorption energy (Eq. 6) [10].


$${lnq}_{e}=lnq_{m}-K_{DR}\varepsilon^{2}$$ (4)

$$\varepsilon^{2}=RTln(1+\frac{1}{C_{e}})$$ (5)

$$E=\frac{1}{\sqrt{2K_{DR}}}$$ (6)

The monolayer saturation capacity (*q*_*m*_) is expressed in units of L/g, while the isotherm constant of adsorption energy (*K*_*DR*_) represents the mean free energy (E) of sorption. Moreover, the application of the Temkin Isotherm (Eq. 7) indicates that the adsorption within the layer diminishes as a result of the interactions between the adsorbent and adsorbate, thereby influencing the coverage.


$$q_{e}=BlnA+BlnC_{e}$$ (7)

In the given context, the symbol B is related to the heat of adsorption and can be calculated using the equation B = RT/b, where T represents the absolute temperature in Kelvin (K), R denotes the gas constant (8.314 J.mol^-1^.K^-1^), and A corresponds to the equilibrium binding constant [10].

In order to establish a wide range of different respects, experimental results were evaluated to the function of the four supposed isotherm models namely, Langmuir, Freundlich, Temkin and D-R. the model parameters investigated from their model equations were calculated and tabulated in Table 3. The basis of model evaluation is based on the assessment of the comparative bases of the correlation coefficient values (*R*^*2*^). The equilibrium data represents a good correlation to Langmuir isotherm as seen from the correlation coefficient values displayed in Table 3. It could be investigated from this suggestion that the sawdust/magnetite adsorption surface is a homogenous type and the acetaminophen adsorption on such surface is signifies to be a monolayer coverage till the saturation of the active sites is occurred. Also, the Frendlich or D-R isotherms are not adequate to describe the adsorption process according to the *R*^*2*^ values in comparison to Langmuir ones. Nevertheless, the lowest correlation coefficient values were linked to the Temkin isotherm model.

**Table 3:** Isotherm parameters for Procion Blue adsorption on different AS-Sorbents.

Isotherm model	Isotherm parameters	Adsorbent				
		M:SD (1:1)	M:SD (1:2)	M:SD (1:3)	M:SD (1:5)	M:SD (2:1)
Langmuir	*a*_*L*_(L/mg)	0.45	0.50	0.51	0.47	0.25
	K_L_	1.92	3.12	1.47	1.47	1.72
	*Q*_*o*_ (mg/g)	6.89	7.64	5.803	3.75	6.69
	*R* ^*2*^	0.98	0.97	0.97	0.87	0.97
Freundlich	*K*_*F*_	2.77	3.26	2.61	1.59	2.57
	*n*	2.11	3.03	1.62	0.78	1.91
	*R* ^*2*^	0.76	0.72	0.78	0.91	0.75
Temkin	*B*(J/mol)	**1.02**	**1.86**	**1.75**	**1.04**	**1.22**
	*A*(L/g)	4.78x10^6^	7.82x10^6^	4.78x10^6^	5.03x10^6^	6.58x10^6^
	*R* ^*2*^	**0.73**	**0.74**	**0.91**	**0.72**	**0.71**
D-R	*q*_*m*_ (mol/g)	**5.73**	**6.35**	**4.53**	**3.88**	**3.42**
	*K*_*DR*_ (mol^2^/J^2^)	1.01x10^-6^	6.058x10^-6^	2.18x10^-6^	4.35x10^-6^	3.22x10^-6^
	*E* (kJ/mol)	**6.50**	**6.33**	**5.49**	**3.33**	**4.39**
	*R* ^*2*^	**0.91**	**0.98**	**0.87**	**0.85**	**0.90**

As tabulated and displayed in Table 3, n value is ranged from 1<n<10 thus the adoption is favorable and n value in the table more than unity, which verifies the favorable adsorption type. Notably, the small values of the mean free energy of adsorption (*E*) represents the physisorption nature of the system hat is weak Van der Waals forces, which is confirmed by the low values of heat of adsorption (*B*).

#### 3.2.5. Kinetic modeling

To further understand the acetaminophen adsorption and to well evaluate the adsorption mechanism and rate controlling stages of sawdust/magnetite, The adsorption system was analyzed using kinetic models. Lagergren’s pseudo-first-order and pseudo-second-order models were utilized to assess the adsorption mechanism. Lagergren’s pseudo-first-order kinetic model (Eq. 8) proposes that a single sorptive site present within the sorbent substance adsorbs one acetaminophen molecule [16].


$$\log\left(q_{e}-q_{t}\right)=\frac{K_{1}}{2.303}t+log(q_{e})$$ (8)

In the given context, *q*_*e*_ represents the amount of acetaminophen adsorbed at equilibrium (mg g^-1^), q_t_ represents the amount of acetaminophen adsorbed at time t (mg g^-1^), and K_1_ represents the rate constant for the pseudo-first-order adsorption. On the other hand, the pseudo-second-order kinetic model (Eq. 9) indicates that the rate of occupation of sorption sites is proportional to the square of the number of available vacant sites [18], providing further support for this concept.

$$\frac{t}{q_{e}}=\frac{1}{K_{2}q_{e}^{2}}+\frac{1}{q_{e}}t$$ (9)

where *K*_*2*_ expressed the adsorption pseudo-second order rate constant.

The adsorption process of acetaminophen on different composite materials was assessed using kinetic models to determine its magnitude and mechanism. The suitability of Lagergren’s pseudo-first-order and pseudo-second-order equations was comparatively evaluated, and the respective parameters for each model are presented in Table 3.

The assessment of each model is conducted by comparing the correlation coefficient values (R^2^) of Lagergren’s pseudo-first-order model and the pseudo-second-order model, as presented in Table 4. The comparison signifies that for all the applied adsorbents, Lagergren’s model is not as a well fit of the adsorption process as the pseudo-second-order model. Such investigation is linked to the excellent *R*^*2*^ of the pseudo-second-order model which is lies in the range of from 0.97 and 0.99. Also, The equilibrium uptake of acetaminophen onto the sawdust/magnetite composite (q_e_), as determined by the pseudo-second-order model, closely approximates the experimental results. However, the model’s calculated adsorption capacity values are slightly lower, indicating that the adsorption system is more intricate than what the model can fully explain. Similar adsorption trends is available in the literature confirming the attained results from the current proposed model indicating a reasonable comparable [5, 10, 22].

**Table 4:** List of kinetic models parameters for acetaminophen adsorption by various adsorbents.

	Model parameters	Adsorbent				
		M:SD (1:1)	M:SD (1:2)	M:SD (1:1)	M:SD (1:2)	M:SD (1:1)
**Lagergren’s Pseudo-first-order**						
	*q*_*e*_ (mg g^-1^))	0.67	0.69	0.43	0.47	0.54
	*k*_*1*_ (min^-1^)×10^−2^	3.71	2.73	1.88	2.54	2.65
	*R* ^*2*^	0.91	0.96	0.88	0.91	0.92
**Pseudo-second-order**						
	*q*_*e*_ (mg g^-1^)	4.66	5.08	2.77	3.27	3.25
	*k*_*2*_ (g mg^-1^ min^-1^)×10^−2^	1.51	1.52	1.71	4.42	1.87
	*R* ^*2*^	0.98	0.97	0.97	0.99	0.98

#### 3.2.6. Beachwood/magnetite composite recyclability

**Fig 11:**
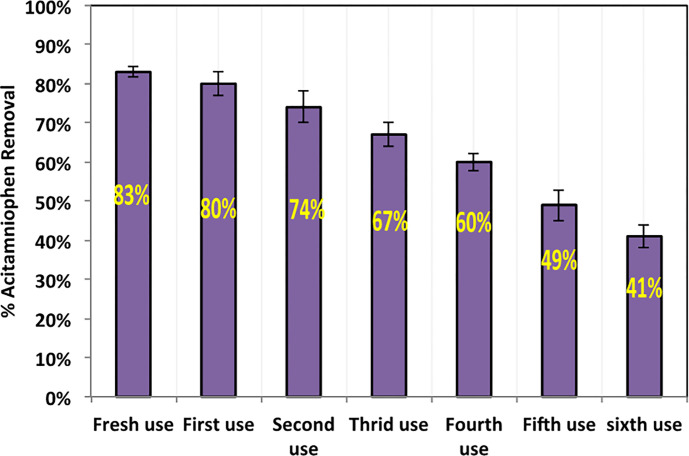
Regeneration for successive use of sawdust/magnetite composite.

For the object of sustainable application, the recyclability of the sawdust/magnetite composite adsorbent is assessed and the data is depicted in Fig 10. Primarily, the sawdust/magnetite composite was recovered and then regenerated after the first use. The substance is regenerated by exploring it for distilled water cleaning and washing for successive times (3 times) in order to remove any saturated acetaminophen molecules and attached to the pores of the sawdust/magnetite surface. Afterwards, the material is tested for four cycles of adsorption–desorption. As displayed in Fig 11, acetaminophen removal efficiency onto the substance was maintained well since the removal capacity is still above 60% after fourth cycles of adsorption compared to 83% for the fresh sawdust/magnetite composite use. This confirms that sawdust/magnetite is an ideal candidate for acetaminophen in aqueous effluent as simulated pharmaceutical discharge treatment applications. However, further use of the catalyst for extra successive cycles, i.e. fifth and sixth cycles results in deterioration in the removal efficiency reached to only 41%. This could be illustrated by the occupied active sites with the pollutant molecules that could not be activated through water washing [15]. It is noteworthy to mention that such results mean the catalyst could be still remove the pollutant even though the efficacy is low.

To assess the environmentally friendly concept of the catalyst for a sustainable competence, the concentration of iron released in the treated effluent is checked and found to be 0.19 mg/L and in comparison to the WHO standard for drinking water regulations (Fe < 0.3 mg/L) it is under the limit [15].

#### 3.2.7. Techno/economic analysis

Globally, academia and industry are working on cost-efficient and feasible systems for a green technology. For full-scale industrial wastewater treatment plants applications, it is essential to attain a crucial concern for both cost analysis and environmental considerations. Not only the optimization of operational parameters affecting the treatment facility, but also implementation and running costs. Thus, at bench scale and pilot scale systems, the chemical and energy costs are applied through the studied systems in the current study. But, when the system is applied for an industrial scale capital cost, equipment maintenance and depreciation costs should be involved. Power consumption is crucial in determining the cost efficiency of the adsorption system since it accounts for a major portion of the operating cost of such processes.

The obtained costs were calculated for the chemicals and electricity used for adsorption process. Approximate cost of power in Egypt (1.20/kWh EGP) is applied in the energy estimation study. Moreover, magnetite preparation cost is approximately EGP 1500 /kg and other chemicals price were almost EGP 32/L. The power consumption per cubic meter of magnetic stirrer was calculated as 100 kW/m3.

The results attained from the current investigation could be scaled up for real industrial wastewater treatment facilities. Thus, it could be summarized that the use of sawdust/magnetite nanoparticles as an adsorbent material in a laboratory results could be an indication for efficient real scale applications. The technology is posses the merits of efficient cost, easily removable and recyclable catalyst. Such merits prove the technology has potential application in real industrial scale. Therefore, updating such technique may be a chance for a green world in the modern era. Thus, future research is still required to significantly explore the real applications. It is noteworthy to mention that imperative financial and environmental costs are forcing the industrial sector to develop alternative management strategies and the answer must be 3 Rs, recycling, reuse and resource recovery.

To assess the environmentally friendly concept of the catalyst for a sustainable competence, the concentration of iron released in the treated effluent is checked and found to be 0.19 mg/L and in comparison to the WHO standard for drinking water regulations (Fe < 0.3 mg/L) it is under the limit.

## 4. Conclusion

Naturally abundant sawdust as a waste augmented with magnetite nanoparticles as a composite prepared by simple treatments and modifications are applied as an adsorbent material. The substance is used as a recyclable sustainable adsorbent material for pharmaceutical simulated waste with acetaminophen elimination. The adsorbent material is prepared in various proportions of sawdust: magnetite and applied as a novel method for removing acetaminophen from aquoues effluent. In the current investigation, the prepared and characterized *Beachwood* sawdust/ magnetite as a low-cost sustainable adsorbent was mixed in various composite proportions and study verified the optimal blend that is corresponding to (1:2) of magnetite and sawdust (M:SD). The experimental parameters were investigated at the contact time of 2 h. Furthermore, 2.0 g/L of adsorbent dose is recorded as the optimal one. The maximum adsorption capacity was recorded at 7.0 mg/g for M:SD (1:2) while the acetaminophen concentration reached to 200 mg/L. The neutral pH of the aqueous media proved to be advantageous for the removal of acetaminophen from wastewater solutions over all adsorbents. The experimental findings indicated that elevated temperatures were not conducive to achieve high adsorption capacities, and the reaction was characterized as an exothermic process. The optimal temperature is recorded at 32°C. Equilibrium sorption isotherm investigation revealed that Langmuir isotherm model well fit the experimental data and the second order kinetics described the adsorption results. Also, a comparative study using various low-cost adsorbents as well as various emerging pollutants based on pharmaceutical effluents from literature have been evaluated and assessed. Recyclability of the catalyst proved the sustainability of the composite that could remove pollutants even the sixth cycylic use after regeneration. Universal abundant waste sawdust makes the treatment method reliable economic and worldwide benign especially once conjugated with the environmentally benign magnetite substance. Further data on real wastewater sample is essential for real scale applications. Also, further work is essential to apply it in a large-scale industrial facility.
